# Supplementary material for: The role of the individual TOM subunits in the association of PINK1 with depolarized mitochondria
Source: J Mol Med (Berl). 2022 Apr 7;100(5):747–62. doi: 10.1007/s00109-022-02191-6 (PMC9110474; doi:10.1007/s00109-022-02191-6)
Supplement: Supplementary file 1 — Supplementary file1 (DOCX 22 KB) [file 109_2022_2191_MOESM1_ESM.docx]

**Table S1. List of cell lines used in this study.**

| **Histology** | **Cell line** | **Growth medium*** | **Source** | **Note** |
| --- | --- | --- | --- | --- |
| Human cervical cancer | HeLa | DMEM* | Kindly provided by Vera Kozjak-Pavlovic, Würzburg, Germany | HeLa cells carrying a doxycycline-inducible shRNA directed against TOM70 (Kozjak-Pavlovic *et al.,* 2007). Uninduced cells were used as control in this study. |
| Human osteosarcoma | U2OS | DMEM* | Kindly provided by Ana J. Garcia-Saéz, Köln, Germany |  |

* DMEM (Dulbecco’s Modified Eagle’s Medium – low glucose with 1000 mg/L, L-glutamine, and sodium bicarbonate supplemented with fetal bovine serum (10% or 20%) and Penicillin-Streptomycin (10,000 units penicillin and 10 mg streptomycin/mL), all purchased from Sigma-Aldrich, Germany.

**Table S2. List of plasmids used in this study.**

| **Plasmid** | **Promoter** | **Coding Sequence** | **Markers** | **Source** |
| --- | --- | --- | --- | --- |
| pRS426-PINK1-eGFP | TPI | Human PINK1 (full-length)-GFP | Amp, URA3 | Lab stock |
| pRS426-PINK1(35-120)-eGFP | TPI | Human PINK1(35-120)-GFP | Amp, URA3 | Lab stock |
| pRS426-PINK1(1-120)-eGFP | TPI | Human PINK1(1-120)-GFP | Amp, URA3 | Lab stock |
| pIVEX1.3-PINK1 | T7 | Human PINK1 full-length | Amp | This study |
| pGEM4-F1β | SP6 | *S. cerevisiae* F1β | Amp | Lab stock |
| pGEM4-hTOM40 | SP6 | Human TOM40 | Amp | Lab stock |
| pSP64-PBR-HA | SP6 | *Mus musculus* PBR-HA | Amp | Lab stock |
| pGEM4-GFP | SP6 | GFP | Amp | Lab stock |
| pGEX4TI-GST-Tom20cd | Tac | *S. cerevisiae* Tom20 cytosolic domain N-terminally tagged with GST | Amp | Lab stock |
| pGEX4TI-GST-Tom70cd | Tac | *S. cerevisiae* Tom70 cytosolic domain N-terminally tagged with GST | Amp | Lab stock |
| pGEX4TI-GST | Tac | GST | Amp | Lab stock |
| pQE60-pSu9-DHFR-His | T5 | *Neurospora Crassa* pSu9 tagged with DHFR and His | Amp | Lab stock |
| pGEX6p1-TcPINK1(1-570) | Tac | *Tribolium castaneum* PINK1(1-570), N-terminally tagged with GST (HRV 3C protease cleavage site between the sequences) | Amp | Lab of Jean-Francois Trempe, Montreal, Canada |
| pGEM4-pSu9-DHFR | SP6 | *Neurospora Crassa* pSu9 tagged with DHFR | Amp | Lab stock |

**Table S3. List of primers used in this study.**

| **Primer name** | **Sequence (5’-3’)** | **Note** |
| --- | --- | --- |
| TNT_hPINK1_For | ATTTAGGTGACACTATAGaagacacggaattcaagccaccatggcggtgcgacaggcgctg | Contains SP6 promoter and Kozak sequence upstream PINK1 (human) starting sequence; used to produce linear DNA for coupled transcription and translation |
| TNT_hPINK1_Rev | cgatccttacagggctgccctccatgagcagagg | Anneals to the 3’ sequence of PINK1 (human), contains the stop codon; used to produce linear DNA for coupled transcription and translation |
| hPINK1_S228A_For | cggcaggtgcgtccagcgaagcc | Mutagenesis S228A |
| hPINK1_S228A_Rev | ggcttcgctggacgcacctgccg | Mutagenesis S228A |
| hPINK1_Q126P_For | ctcggcctgtccggagatccagg | Mutagenesis Q126P |
| hPINK1_Q126P_Rev | cctggatctccggacaggccgag | Mutagenesis Q126P |
| TNT_TcPINK1_For | ATTTAGGTGACACTATAGaagacacggaattcaagccaccATGAGCGTTCGTGCAGTTGG | Contains SP6 promoter and Kozak sequence upstream PINK1 (*Tribolium castaneum*) starting sequence; used to produce linear DNA for coupled transcription and translation |
| TNT_TcPINK1_Rev | cttggatcCttaATCCAGTTCCGGCAGG | Anneals to the 3’ sequence of PINK1 (*Tribolium castaneum*), contains the stop codon; used to produce linear DNA for coupled transcription and translation |
| TNT_hPINK1(1-120)-eGFP_For | ATTTAGGTGACACTATAGaagacacggaattcaagccaccatggcggtgcgacaggcgctg | Contains SP6 promoter and Kozak sequence upstream PINK1 (human) starting sequence; used to produce linear DNA for coupled transcription and translation |
| TNT_hPINK1(1-120)-eGFP_Rev | Gcttcgagcgtcccaaaaccttctcaagcaagg | Reverse primer for hPINK1(1-120)-eGFP; used to produce linear DNA for coupled transcription and translation |
| TNT_hPINK1(35-120)-eGFP_For | ATTTAGGTGACACTATAGaagacacggaattcaagccaccatgggcccggcggcgggctg | Contains SP6 promoter and Kozak sequence upstream PINK1 (human) starting sequence; used to produce linear DNA for coupled transcription and translation |
| TNT_hPINK1(35-120)-eGFP_Rev | Gcttcgagcgtcccaaaaccttctcaagcaagg | Reverse primer for hPINK1(35-120)-eGFP; used to produce linear DNA for coupled transcription and translation |

**Table S4. List of antibodies used in this study.**

| **Antibodies** | **Dilution** | **Source** |
| --- | --- | --- |
| Mouse monoclonal Anti-VDAC1 | 1:500 | ab14734, abcam |
| Rabbit polyclonal Anti-TOMM22/TOM22 | 1:500 | ab246862, abcam |
| Rabbit polyclonal anti-Human Cytochrome b-c1 complex subunit 8 | 1:500 | MBS715150, MyBioSource |
| Mouse monoclonal anti-TOM70 | 1:500 | sc-390545, Santa Cruz Biotechnology |
| Mouse monoclonal anti-TOM40 | 1:500 | sc-365467, Santa Cruz Biotechnology |
| Rabbit polyclonal anti-TOM40 | 1:500 | sc-11414, Santa Cruz Biotechnology |
| Mouse monoclonal anti-TOM20 | 1:500 | sc-17764, Santa Cruz Biotechnology |
| Mouse monoclonal anti-ATP5A | 1:500 | ab14748, abcam |
